# The Moral and Gender Implications of Measures Used to Modulate the Mobility of People With Dementia Living in Residential Care Environments: A Scoping Review

**DOI:** 10.1093/geront/gnad071

**Published:** 2023-06-18

**Authors:** Jodi Sturge, Sarah Janus, Sytse Zuidema, Brenda Frederiks, Mark Schweda, Elleke Landeweer

**Affiliations:** Department of General Practice and Elderly Care Medicine, University of Groningen, University Medical Center Groningen, Groningen, The Netherlands; Department of General Practice and Elderly Care Medicine, University of Groningen, University Medical Center Groningen, Groningen, The Netherlands; Department of General Practice and Elderly Care Medicine, University of Groningen, University Medical Center Groningen, Groningen, The Netherlands; Department of Ethics, Law and Humanities, University Medical Centre Amsterdam, Amsterdam, The Netherlands; Division of Ethics in Medicine, Department of Health Services Research, School of Medicine and Health Sciences, Carl von Ossietzky University of Oldenburg, Oldenburg, Germany; Department of General Practice and Elderly Care Medicine, University of Groningen, University Medical Center Groningen, Groningen, The Netherlands

**Keywords:** Dementia care, Human rights, Open-door policy, Sex and gender, Transformation

## Abstract

**Background and Objectives:**

Policies and measures often restrict the mobility of people with dementia living in residential care environments to protect them from harm. However, such measures can violate human rights and affect the quality of life. This review aims to summarize the literature on what is known about measures used to modulate the life-space mobility of residents with dementia living in a residential care environment. Furthermore, moral and sex and gender considerations were explored.

**Research Design and Methods:**

A scoping review framework was referenced to summarize the literature. A total of 5 databases were searched: PubMed, Embase, CINAHL, SCOPUS, and Web of Science. The studies for eligibility using the Rayyan screening tool.

**Results:**

A total of 30 articles met the inclusion criteria. A narrative description of the findings of the articles is presented across 3 themes: (1) measures and strategies used to modulate the life-space mobility; (2) moral aspects; and (3) sex and gender considerations.

**Discussion and Implications:**

Various measures are used to modulate the life-space mobility of people with dementia living in residential care facilities. Research exploring the sex and gender differences of people with dementia is lacking. With a focus on human rights and quality of life, measures used to restrict or support mobility must support the diverse needs, capacity, and dignity of people with dementia. Noting the capacity and diversity of people with dementia will require society and public space to adopt strategies that promote safety and mobility to support the quality of life of people with dementia.

## Background and Objectives

People with dementia are often considered to be at risk of harm due to a decline in cognition. To support a safe environment, many specialized dementia care environments lock their doors to prevent wandering and protect residents from eloping, getting lost, or death (Duffy & Hallahan, 2019). The prevalent focus on physical safety in dementia care has been contested. Such policies limit the individual freedom of movement of all residents, not only those at risk of harm or getting lost (Frederiks, 2020). Additionally, these policies are seen to interfere with the rights of residents under the United Nations Convention on the Rights of Persons with Disabilities, the European Convention on Human Rights, and other international laws (Butchard & Kinderman, 2019; Cahill, 2022; De Sabbata, 2020; Smith & Sullivan, 2012). Meanwhile, several countries (i.e., Canada, England, Finland, Germany, the Netherlands, Norway, and Australia) continue to debate when and to what extent restricting the freedom of movement is morally legitimate and to what extent risk management is considered to be good care (Dixon et al., 2020; Driessen, 2020; Landeweer et al., 2021; Lowndes et al., 2021; Niemeijer et al., 2015; Piirainen et al., 2021; Steele et al., 2019; Tufford et al., 2018). Such discussions have led to more dementia care environments with free access throughout and beyond the main doors of the facility.

Although restrictive measures are generally used to prevent harm and provide a safe environment, using some measures can affect residents’ mobility and quality of life. Mobility, described as “the ability to move oneself within environments that expand from one’s home to the neighborhood and regions beyond,” is a critical component of healthy aging (Webber et al., 2010, p. 444). Understanding the mobility of older adults with cognitive decline can provide valuable insight into decision-making and choices essential for a sense of agency and well-being (Sturge et al., 2020). Life-space mobility is a concept used to contextualize and assess the mobility of older adults across zones structured around a central geographic point (Johnson et al., 2020; Peel et al., 2005; Sverdrup et al., 2021). For residents living in residential care, life-space mobility is understood across four zones; the patient room (central point), outside the room but within the unit, outside the unit but within the facility, and outside the facility (Tinetti & Ginter, 1990). Residential care environments can both support and restrict life-space mobility. Often, such measures are universally applied to the entire unit and do not reflect individual needs or the level of risk of elopement.

The purpose of this review was to gather and summarize research on measures used to restrict or support the life-space mobility of people with dementia living in residential care. Additionally, we explored the moral considerations and risks as well as sex and gender aspects. Identifying care needs based on sex and gender is fundamental where, dementia affects women more than men in prevalence and severity (Carter et al., 2012). Women tend to have a poorer physical function and more risk of mobility disabilities than men (Webber et al., 2010; Zunzunegui et al., 2015), therefore may have a lower risk of eloping. However, older men with dementia in residential care facilities tend to be at higher risk of social exclusion (Bartlett, 2007; Bartlett et al., 2018), exhibit aggressive behavior (Lövheim et al., 2009), and express intimacy needs differently (Roelofs et al., 2015). Despite these differences, there is limited knowledge of how sex and gender are reflected in dementia care (Sindi et al., 2021). This combined knowledge is timely because it aligns with the growing international attention on the human rights and quality of life of people with dementia living in residential care environments (Beerens et al., 2013; Steele, Carr, et al., 2020, Steele, Swaffer, et al., 2020).

## Research Design and Methods

This scoping review refers to the methodological framework developed by Arksey & O’Malley (2005) and later enhanced by Peters et al. (2015) and Tricco et al. (2018). No similar evidence synthesis was identified that explored this topic, and the purpose was to identify key characteristics and knowledge gaps. Therefore, based on the decision tree developed by Pollock et al. (2021) and methodological guidance for a quality review (Heyn et al., 2019), it was decided to conduct a scoping review instead of a systematic review. To support the rigor of the search, we referred to the methodological guidelines of the Preferred Reporting Items for Systematic Reviews and Meta-Analysis extension for Scoping Reviews (PRISMA-ScR; (Tricco et al., 2018; Supplementary Table 1). This review was registered with Open Science Framework (https://osf.io/8cdq7).

### Stage 1: Identifying the Research Questions

The guiding research questions were:

1. What measures and strategies are used to modulate the life-space mobility of people with dementia living in residential care environments?
2. What are the moral considerations and issues associated with such measures?
3. How are the sex and gender differences of people with dementia reflected in such measures?

### Stage 2: Identifying the Relevant Studies

The search strategy and database selection were defined and refined by an information specialist at the University Medical Center Groningen. The search included a combination of MeSH terms and free text terms (Supplementary Table 2) in a Boolean search format using AND, OR, and NOT operators. As advised by the information specialist, the following electronic databases were searched: PubMed, Embase, CINAHL, SCOPUS, and Web of Science. The search strategy was developed in PubMed (Supplementary Figure 1) and later reformatted to other databases. No date limits were defined to capture a historical perspective of measures used. One author (J. Sturge) and an information specialist performed the initial search. Sequential searches were conducted by one author (J. Sturge). All searches were completed in May 2022. In total, 6,071 articles were identified.

### Step 3: Study Selection

The population, concept, and context (PCC) mnemonic (Table 1) was used to guide the focus inclusion criteria of this review (Peters et al., 2022).

**Table 1.** Inclusion and Exclusion Criteria Based on Population Concept and Context

PCC framework	Inclusion criteria	Exclusion criteria
Population	Persons with dementia	A focus on persons without dementia
Concept	Measures used to prevent people with dementia from exiting designated areas of a residential care environment or leaving the facility from the main door	Measures used to mitigate other behaviors such as restlessness and agitation
Context	Focus on institutional care, such as residential care facilities	Home environments

*Note:* PCC = population, concept, and context.

Furthermore, without restrictions on the date, articles had to meet the following inclusion criteria:

- Described a residential care environment for people living with dementia;
- Described a feature or policy that restricts or supports the mobility of residents based on empirical data;
- Peer-reviewed articles or conference proceedings;
- Published in Dutch, English, or German.

**Figure 1.**
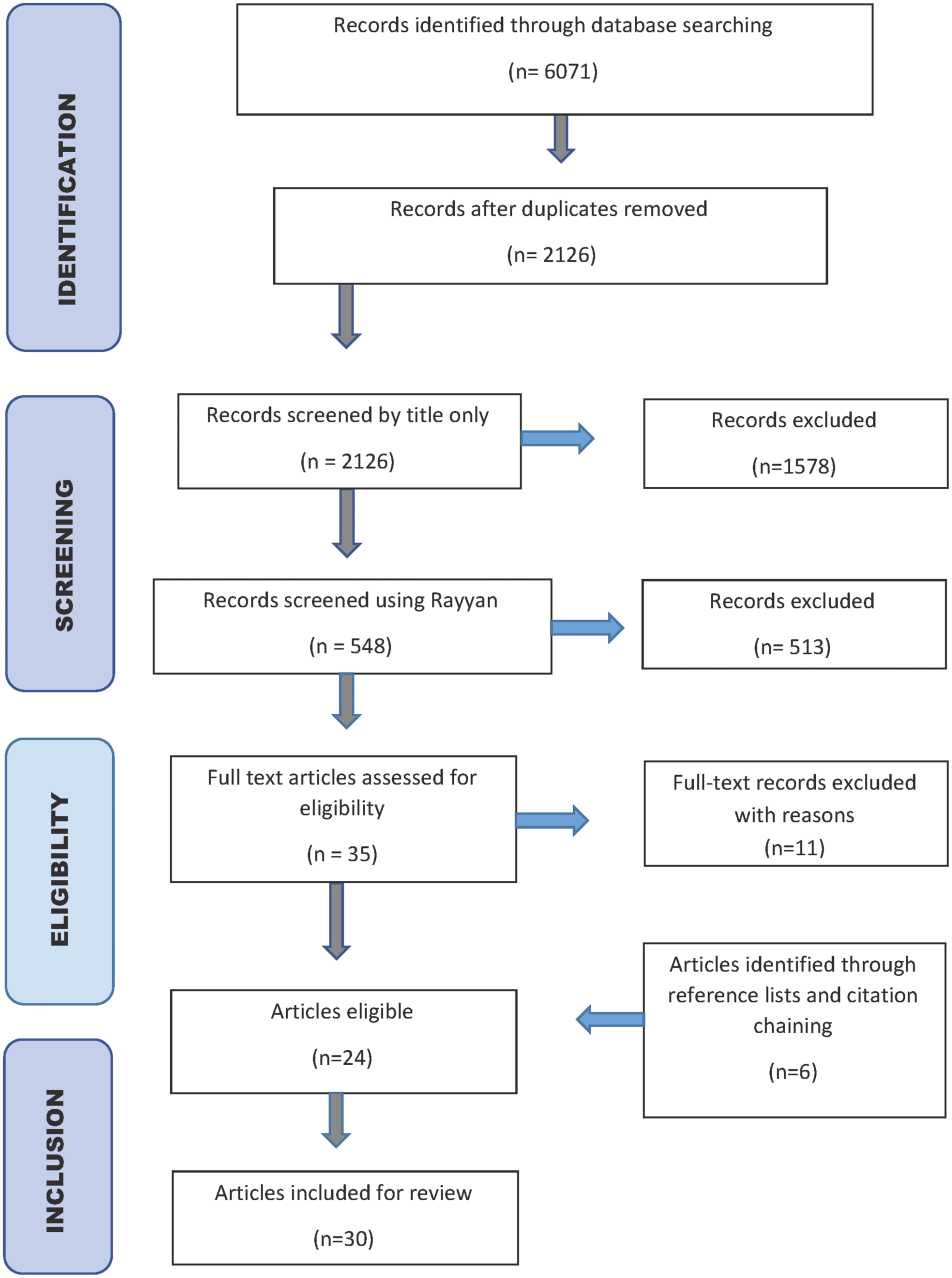
Search strategy.

The search results were imported into reference software EndNote X9 where duplicates were removed, resulting in 2,126 articles. One author (J. Sturge) initially screened the article titles, resulting in 548 potentially eligible articles left for closer consideration. The titles and abstracts of the articles were imported into the Rayyan screening tool and screened independently using the text mining function (Olofsson et al., 2017). Three authors (J. Sturge, S. Janus, and E. Landeweer) screened the titles and abstracts of the articles, referring to the PCC and inclusion criteria. Using the blind setting in Rayyan, one author (J. Sturge) reviewed articles, and two authors (S. Janus and E. Landeweer) divided the list equally. Discrepancies between the reviewer’s decisions were identified in the inclusion decision tab in Rayyan, and decision discrepancies were discussed. Reasons for exclusion included no description of a measure to restrict or support mobility or a hospital environment. A short list of selected articles was exported into Excel (.csv file). The references of the selected articles were checked using backward reference list checking and the Google Scholar “cited by” function to forward check the selected relevant studies. The final step was a full-text review of the selected articles completed by two reviewers (J. Sturge/E. Landeweer). The search strategy is shown in Figure 1.

### Step 4: Charting the Data

The selected articles were charted in a table format suggested by Arksey and O’Malley (2005) with information on the lead author, year of publication, study location, study aim, research method, research population, sex and gender description, and measures used to restrict or support mobility (Table 2).

**Table 2.** Summary of Included Articles

Authors (year) country	Research method	Research population	Sex/gender description of persons with dementia	Measures used to prevent exiting or support autonomy
Aud (2004) United States	Qualitative	People with dementia who have eloped from a long-term care environment (*n* = 62)	Incidents with women (*n* = 31) Incidents with men (*n* = 27) Incidents with gender not specified (*n* = 4)	• Alarms on exit doors (keypads and panic bars) • Wearable sensors for patients (wrist bracelet)
Boumans et al. (2022) The Netherlands	Qualitative	Residents with dementia (*n* = 73) Residents without dementia (*n* = 37) Full-time care staff (*n* = 133)	Not described	• Care watches • Staff training/hospitable approach to care • Tags in clothes
Te Boekhorst et al (2013) The Netherlands	Quantitative	Residents with dementia living in nursing homes with surveillance technology (*n* = 170) or physical restraints (*n* = 22)	Female (*n* = 123) Male (*n* = 69)	Surveillance technologies: • cameras, • acoustic monitoring systems, • chips worn in clothing or shoes that control doors • tracking chips with GPS • (Inactivity sensors, movement sensors in beds or chairs, door sensors, and bed pressure sensors)^a^ Physical restraints^a^
Chafetz (1990) United States	Quantitative	People with dementia (*n* = 30)	Women (*n* = 28) Men (*n* = 2)	• Visual-grid made with tap on the floor
Ciofi et al. (2022) United States	Qualitative	Residents with dementia (*n* = 33) Informal care partners (*n* = 40) Formal care partners (*n* = 60)	Female (*n* = 8) Male (*n* = 4) Transgender (*n* = 1)	• Alarm code • Escorted visited beyond care environment • Outdoor seating • Garden spaces and courtyard • Walking paths • Planned activities beyond the care environment
Cohen-Mansfield and Werner (1998) United States	Mixed method	People with dementia (*n* = 27)	Female (*n* = 21) Male (*n* = 6)	• Two enhanced environments (nature and home-like setting murals) with benches facing the scene.
Cohen-Mansfield and Werner (1999) United States	Quantitative	Nursing home staff (*n* = 320)	Not described	• Activities **• **Design features in outdoor spaces
Detweiler et al. (2008) ^b^ United States	Qualitative	Residents in a nursing home unit (*n* = 34)	Male (*n* = 34)	• Activities in wander garden • Walkways • Wander garden
Dickinson and McLain-Kark (1998) United States	Qualitative	Residents of a dementia care unit (*n* = 8)	Female (*n* = 3) Male (*n* = 5)	• A cloth panel covering the panic bar of the fire exit door. • A mini-blind covering the panic bar of the fire exit door.
Dickinson et al. (1995) United States	Qualitative	Ambulatory residents of a dementia care unit with histories of exiting behavior (*n* = 7)	Females (*n* = 2) Males (*n* = 5)	• A cloth panel covering the panic bar of the fire exit door. • A mini-blind covering the panic bar of the fire exit door. • Both a mini-blind and cloth panel covering the panic bar of the fire exit door. • No barrier
Evans et al. (2018) ^b^ United Kingdom	Qualitative	Managers from care homes offering dementia car(*n* = 18)	Females (*n* = 16) Males (*n* = 2)	• Locked doors • Keypads • Gardens • Outdoor spaces
Favez et al. (2022) Switzerland	Quantitative	Residents of nursing homes (*n* = 6,149)^c^ Staff of nursing homes (*n* not specified)	Females (*n* = 4,341) Males (*n* = 1,808)	• Surveillance technologies • Pressure detection mats • Cameras • Electronic system to control the ability to open doors • Electronic bracelets
Graham and Fabricius (2018) Canada	Qualitative	Residents of secured dementia care unit with veteran status (*n* = 36) Residents of secured dementia care unit with non-veteran status (*n* = 40) Staff (*n* not specified)	Veteran unit: Female (*n* = 3) Males (*n* = 33) Non-veteran unit: Female (*n* = 26) Male (*n* = 14)	• Mural designs created by residents on exit doors
Graham and Fabricius (2021) Canada	Qualitative	Residents of a secure specialized behavior unit (*n* = 20)	Female and male residents (sex breakdown not specified)	• Interactive door mural with magnets of objects for a magnetic bookcase.
Hall et al. (2017) England	Qualitative	Residents (*n* = 9) Relatives (*n* = 9) Staff (*n* = 24)	Not specified, but stated the majority of the sample was female	• Alarms • Activity trackers: • Location-based systems • Wearable fobs • Bed-monitoring technology • (Bed sensors and pressure mats)
Kincaid and Peacock (2003) United States	Quantitative	Residents of a special care unit (*n* = 17)	Women (*n* = 10) Men (*n* = 2) Gender not specified (*n* = 5)	• Wall murals on the entrance/exit doorway
Lowndes et al. (2021) Canada, Norway, and Germany	Qualitative	Residents of segregated dementia units (*n* = 7) Management, staff, volunteers, students, families, and resident across 13 sites	Not specified	• Engaging activities • Gardens • Offsite activities • Sensory rooms
Margot-Cattin and Nygård (2006) Switzerland	Qualitative	Residents of a geriatric psychiatry unit (*n* = 15) Staff members (*n* = not described) Residents of the unit (*n* = not described)	Not explicitly described but mention of two females and one male were observed more closely	• A wearable chip card to lock and unlock doors
Mazzei et al. (2014) Canada	Qualitative	Residents of an acute geriatric psychiatry care (*n* = 6)	Women (*n* = 2) Men (*n* = 4)	• Camouflage murals on exit doorways to reduce door testing • Circular as opposed to a linear, wandering path • of the residents rather than four-bed wards • Outdoor patio for residents • Private bedrooms with adjoining bathrooms for the majority
Müller et al. (2010) Germany	Qualitative	Family caregivers and professional care-givers	Not explicitly described but description of sex/gender behavior	• GPS locating system
Niemeijer et al. (2015) ^b^ The Netherlands	Qualitative	Residents of a dementia special care unit of a nursing home (*n* = 43) A residential care facility for people with intellectual disabilities (CFID) (*n* = 24)^d^	Not specified	Surveillance technologies: • Acoustic sensors • Bracelets • Video surveillance
Øye and Jacobsen (2020) Norway	Mixed method	Nursing home staff (*n* = 43)	Not described	• Diverting attention • Fake bus stops • White lies
Sandberg et al. (2022) Finland	Qualitative	Residents of nursing homes (*n* = 15) Family members (*n* = 27) Nurses (*n* = 22)	Not described	• Closed doors • Gardens
Steele, Carr et al. (2020) ^b^ Australia	Qualitative	People living with dementia (*n* = 15) Care partners (*n* = 10) Care home professionals (*n* = 15) Lawyers and advocates (*n* = 10)	Not described	• Escorted outings • Fences • Gardens • Locked doors
Steele, Swaffer et al. (2020) Australia	Qualitative	People living with dementia (*n* = 15) Care partners (*n* = 10) Care home professionals (*n* = 15) Lawyers and advocates (*n* = 10)	Not described	A general description of secured dementia care environments
Tufford et al. (2018) ^b^ Canada, Norway and Germany	Qualitative	Residential care management, health providers, support staff, informal care providers, union representatives, residents and family members	Not described	• Alarms • Closed and open-door units • Elevators • Locks on doors • Outdoor spaces
van Hecke et al. (2019) ^b^ Belgium	Qualitative	Residents (*n* = 4) Staff members (*n* = 5)	Residents Female (*n* = 4) Staff Female (*n* = 4) Male (*n* = 1)	• Architecture • Codes • Elevators • Gardens
Varshawsky and Traynor (2021) Australia	Quantitative	Individuals living with dementia (*n* = 9)	Not described	• Adhesive posters placed on bedroom doors
Wigg, 2010^b^ United States	Qualitative	Individuals living with dementia (*n* = 30)	Not explicitly described but mentioned a gender ratio of one male to three females	• Garden spaces • Large picture windows • Locked doors • Motion dectors • Open-door policy
Zwijsen et al. (2012) The Netherlands	Qualitative	Care professions including physicians, and managers (*n* = 9)	Not described	• Acoustic monitoring • Chips worn in clothing • Door sensors • GPS locating system

*Notes*:

^a^Findings describing physical restraints were excluded from this review.

^b^Article was included in the van Liempd et al. (2022) review.

^c^People with dementia not specified, however, 209 of 292 of the nursing home units offered dementia-specific services.

^d^Findings for this population group were excluded from this review.

### Step 5: Collating, Summarizing, and Reporting the Results

Using a descriptive and qualitative approach, the findings of the studies were extracted and grouped based on common characteristics and narrative descriptions of the results of the selected articles that align with the research questions.

## Results

### Characteristics of the Selected Articles

A total of 30 articles were identified, with publication dates ranging from 1990 to 2022. The studies were based on results from Western countries, including the United States (*n* = 10), Europe (*n* = 9), Canada (*n* = 3), United Kingdom (*n* = 3), Australia (*n* = 3), and multi-country comparison (*n* = 2). Most of the studies were based on qualitative research methods (*n* = 22). Studies were published in journals from a range of disciplines, including nursing, ethics, sociology, and architecture. The study populations ranged from people with dementia (*n* = 14), solely care professionals (*n* = 4), or a mix of people with dementia, family, and staff (*n* = 11). Just over half of the care environments were described as nursing homes or dementia care units, and assisted living environments were only included if they were specific for people with dementia and described the function of a closed door (Ciofi et al., 2022). Most studies (*n* = 24) were conducted in a closed-door environment that prevented exiting or trespassing beyond a designated unit. Six articles, with the majority published in the past five years, described an open-door policy (Boumans et al., 2022; Lowndes et al., 2021; Øye and Jacobsen, 2020; Steele, Carr, et al., 2020; Tufford et al., 2018; Wigg, 2010). This observation may relate to changes in moral attitudes with open-door policies.

#### Measures and strategies used to modulate the life-space mobility of people with dementia living in residential care environments

*Alarms and access-controlled exits.—*Several studies describe how alarms and access-controlled keypads are used to lock doors or control elevator use when residents are attempting to leave the environment (Aud, 2004; Ciofi et al., 2022; Cohen-Mansfield & Werner, 1999; Evans et al., 2018; Favez et al., 2022; Graham & Fabricius, 2018; Hall et al., 2017; Kincaid & Peacock, 2003; Margot-Cattin & Nygård, 2006; Niemeijer et al., 2015; Te Boekhorst et al., 2013; Tufford et al., 2018; van Hecke et al., 2019; Wigg, 2010). In some environments, transmitters or trackers were worn (e.g., electronic bracelets) or sewn into resident’s garments to trigger an alarm to prevent residents from wandering beyond the designated door (Boumans et al., 2022; Favez et al., 2022; Margot-Cattin & Nygård, 2006; Niemeijer et al., 2015; Te Boekhorst et al., 2013; Zwijsen et al., 2012).

*Camouflaged exits and animated features.—*Residents with dementia can be distracted from leaving the care environment with the use of several measures. Staff may use white lies to “lure and trick” residents to convince them to stay in the environment at the (fake) bus stop (Øye & Jacobsen, 2020, p. 194). A typical physical measure is to disguise exit doors with blinds, cloth, or murals. Such modifications can range from simple visual approaches (i.e., tape grid placed before an exit door; Chafetz, 1990) to more sensory-enhanced environments (Cohen-Mansfield & Werner, 1998; Graham & Fabricius, 2018, 2021). The type of mural designs varies from cartoon-like aquarium scenes (Kincaid & Peacock, 2003), bookcases (Mazzei et al., 2014), or nature or home setting scenes (Cohen-Mansfield & Werner, 1998). Murals tend to be created based on photos, stickers, or adhesive posters illustrating features such as a front door (van Hecke et al., 2019; Varshawsky & Traynor, 2021). Murals can be interactive with magnetic objects for residents to gather, carry, and rearrange (Graham & Fabricius, 2021) or multi-sensory with audio features such as music, nature sounds, and scents (Cohen-Mansfield and Werner, 1998).

*Assistive technology.—*Implementing assistive technology can allow residents with dementia to have more freedom and movement within designated spaces and provide opportunities to visit friends in the building and assist them in navigating back to their rooms (Boumans et al., 2022; Margot-Cattin & Nygård, 2006; Niemeijer et al., 2015; Zwijsen et al., 2012). Surveillance technology can provide nursing staff with a sense of assurance in knowing where the residents are, especially when understaffed (Hall et al., 2017; Margot-Cattin & Nygård, 2006), and rely less on physical restraints (Favez et al., 2022; Te Boekhorst et al., 2013). Such technology can track residents’ movement beyond the direct care environment. Devices, such as GPS, can be used to track residents and support their autonomy. It can be a solution to not having enough staff or volunteers to accompany residents who want to leave the facility (Müller et al., 2010; Niemeijer et al., 2015). Although the resident can benefit from exiting, so can the care provider in terms of an equalized relationship and interpersonal interaction (Wigg, 2010).

*Outdoor space, walking loops, and gardens.—*Several care facilities have designated outdoor spaces to enhance the sensory stimulation, mobility, and well-being of residents with dementia (Cohen-Mansfield & Werner, 1999). Outdoor areas can include looped pathways, gardens or points of reference within the built environment (Ciofi et al., 2022; Detweiler et al., 2008; Margot-Cattin & Nygård, 2006; Mazzei et al., 2014; Sandberg et al., 2022; van Hecke et al., 2019). Enclosed garden spaces are described by several articles as a positive addition to the care environment (e.g., Cohen-Mansfield & Werner, 1999; Detweiler et al., 2008; Lowndes et al., 2021; Steele, Carr, et al., 2020). The spaces allow residents to leave a locked unit and access nature, reducing the risk of resident elopement (Detweiler et al., 2008). Furthermore, additional features such as lawn furniture, interaction with birds or pets, and extracurricular activities can make these spaces more enjoyable (Cohen-Mansfield & Werner, 1999).

*Special events and activities.—*Special activities organized beyond the immediate care environment, such as outings or a food truck event described by Ciofi et al. (2022), can alleviate residents’ impulse to leave the facility to get home. Furthermore, riding in a car or bus (Lowndes et al., 2021) or leaving the residential environment with an escort (Steele, Swaffer, et al., 2020) is beneficial for some residents. Such excursions may allow people with dementia to engage with broader communities, including others without dementia (Ciofi et al., 2022).

*Architecture and design.—*The overall architecture of the care environment can support movement and wandering (Wigg, 2010) and provide residents with a sense of freedom and mobility. For example, unique bedroom door designs with a range illustrated of architectural features, such as door knockers or letterboxes, can help residents with wayfinding and visual recognition (Varshawsky & Traynor, 2021). The building structure and floor plan can support residents’ orientation, and large windows can provide views of the outdoor environment. Furthermore, when there are children or animals outside the window, these views can provide a sense of freedom and contribute to the quality of life of residents (Tufford et al., 2018; van Hecke et al., 2019; Wigg, 2010).

#### Moral aspects

*Reliability and staff availability.—*Alarms or key-coded doors are not always a reliable measure to prevent exit. In some circumstances, the alarm system did not work, or the staff needed to be trained to operate the alarm. Staff appeared overburdened by frequent false alarms and decided to turn the alarm off (Aud, 2004; Hall et al., 2017; Niemeijer et al., 2015). To ensure that technology is used as intended, technology should also be modified (e.g., the time setting of an opened door or location of the sensor) to respond to the resident’s pace (Margot-Cattin & Nygård, 2006).

Even in environments with alarms on the main door, people with dementia can tailgate behind persons with technology or exchange the devices between residents to leave the locked environment (Aud; 2004; Ciofi et al., 2022; Margot-Cattin & Nygård, 2006; Wigg, 2010; Zwijsen et al., 2012). Furthermore, residents who were aware of the alarms interfered by removing the devices or entering the code posted on the exit door (Aud, 2004; Margot-Cattin & Nygård, 2006). Residents encountering a locked door may want to leave the environment but are unaware that a code is required. Such encounters can negatively affect their quality of life, resulting in agitation, depressive behaviors of withdrawal, and other problematic behaviors (Ciofi et al., 2022; Kincaid & Peacock, 2003; Sandberg et al., 2022; Tufford et al., 2018; Wigg, 2010).

Surveillance technology is only reliable if the staff can operate and monitor it. There can be additional challenges, such as adequately placing the technology on the resident to ensure it works (Margot-Cattin & Nygård, 2006). Furthermore, several studies mention moral concerns about residents’ privacy when using technology to track behavior (Hall et al., 2017; Margot-Cattin & Nygård, 2006; Niemeijer et al., 2015). Also, surveillance technology may only benefit those who can move without help (Te Boekhorst et al., 2013). The accommodation requires surveillance or observation, but there is still a degree of pathologization (Steele, Swaffer, et al., 2020). Some residents resist wearing trackable technology such as bracelets (Niemeijer et al., 2015) because they feel stigmatized. Also, using technology to leave the environment only available to residents willing to pay for the device (Boumans et al., 2022) makes this option inequitable to all residents.

Even if care environments have enclosed gardens, residents do not always have direct access to these gardens (van Hecke et al., 2019); access to garden spaces is often limited and controlled by staff through a locked door or keypad (Ciofi et al., 2022; Evans et al., 2018; Tufford et al., 2018). Access can be limited during a particular time window (Cohen-Mansfield & Werner, 1999; Detweiler et al., 2008) or dependent on the weather or season (Ciofi et al., 2022; Evans et al., 2018; Tufford et al., 2018; Wigg, 2010). Gardens are often closed due to insufficient staff or volunteers to monitor residents or respond if a resident falls (Lowndes et al., 2021; Øye & Jacobsen, 2020; Sandberg et al., 2022). Gardens are also not a feature for every resident. In some articles, the spaces were not often used and some residents were not used to going outside (Cohen-Mansfield & Werner, 1999; Müller et al., 2010; van Hecke et al., 2019). Furthermore, not all residents were engaged in activities, leaving them marginalized or excluded from opportunities to engage in activities beyond the immediate care setting (Ciofi et al., 2022; Lowndes et al., 2021; Steele, Swaffer, et al., 2020). In addition, staffing shortages can affect the frequency and sustainability of additional activities or events (Tufford et al., 2018). Even with prior planning, residents’ reactions to offsite activities can be unpredictable and distressing, resulting in residents becoming disoriented or challenging to manage (Ciofi et al., 2022).

*Deceptive and agitating features.—*Features such as murals can also affect residents’ dignity. Residents can become restless and agitated when they know they are being deceived into staying in the environment and are consequently drawn to the door (Dickinson & McLain-Kark, 1998; Dickinson et al.,1995; Evans et al., 2018). As the van Hecke et al. (2019) study describes, a door with no visible handles and flower stickers on a white surface extending to the ceiling can resemble a wall. However, residents fixate on the area where they could recognize the actual function as an entrance/exit where staff or visitors could open it. This type of interaction is similarly described in another study where residents were still cognitively aware of people coming in and out through the “murals” despite their bookcase camouflage (Mazzei et al., 2014). In addition, murals can be patronizing, outdated, and not engaging for residents. For example, mimicking the outdoors with objects (garden or beach theme), buckets and spades (Evans et al., 2018), or decorative fabrics commonly used in children’s daycare centers (Tufford et al., 2018) may not reflect the needs or interests of residents. Similarly, some features can become dated or dull. In the Sandberg et al. (2022, pp. 18) study, a participant expressed a sense of boredom using the feature: “And one walks around in circles in the inner yard. I have thought, how long still? If I live to a hundred years, I still have sixteen years left.” Furthermore, not all residents want to be accompanied constantly, which offends a sense of freedom (Müller et al., 2010).

*Health and safety design regulations.—*The design and the architecture of a facility can be limited by government regulations and standards, making the environments stigmatizing and institutional (Tufford et al., 2018). The placement of windows can create a zoo-like effect where residents feel that everything happening beyond the window is unreachable, which causes confusion or agitation (Evans et al., 2018; van Hecke et al., 2019). Furthermore, it is typical that high fences enclose the facility or garden spaces (Kincaid & Peacock, 2003; Sandberg et al., 2022; Steele, Carr, et al., 2020, Steele, Swaffer, et al., 2020). These structures may support a larger circle of movement but do not reduce the sense of confinement. Also, the layout of the facility can make it challenging to navigate the site (Tufford et al., 2018) and access all areas. As seen in Evans et al. (2018) and van Hecke et al. (2019) studies, only the residents on the ground floor had independent access to the garden spaces or residents with access to an elevator.

The design of murals also needs to consider the safety of the environment. For instance, the design should be flat, not shiny or reflective, and doors and handles should be blended visually (Dickinson et al., 1995; Graham & Fabricius, 2018; Kincaid & Peacock, 2003). To ensure health and safety, regulatory bodies in some countries (e.g., Canada and the United States), such as fire departments and licensing inspectors, must approve the designs and access to the exit in case of an emergency. For instance, when a mural is used to camouflage a door exit, signs often need to be displayed over the doors, and windows need to remain functioning with transparency to allow visualization to avoid the risk of colliding with people on the other side of the door.

#### Sex and gender considerations

The sex and gender of the research samples were described in 14 articles. Seven articles included samples that were either exclusively or majority women (Aud, 2004; Chafetz, 1990; Cohen-Mansfield & Werner, 1998; Graham & Fabricius, 2018; Hall, Wilson, Stanmore, & Todd, 2017; Kincaid & Peacock, 2003; Te Boekhorst et al., 2013; van Hecke et al., 2019) which reflects the majority of residents with dementia living in residential care. Only one article included a sample of only men (Detweiler et al., 2008), and one study included a transgender participant (Ciofi et al., 2022).

Only five articles described sex and gender differences. Müller et al. (2010) described differences in managing the behavior of men compared with women stating that women with dementia are often more “manageable” (Müller et al., pp. 80). Tufford et al. (2018) noted that most of the work in care homes is done by women, which results in a gender-specific dynamic of staff. No study described tailoring measures based on the sex and gender needs of the residents. However, Lowndes et al. (2021) noted limited opportunities to engage residents in gender-specific activities. The study described how most activities in care environments are gender-neutral or aligned with mostly female interests such as bingo, crafts, knitting, or cooking. However, when activities are created to meet men’s needs and interests, such as men’s club, beer night with card playing, fishing, or workshop areas, male residents enthusiastically attend the activities. These findings relate to Graham and Fabricius (2018), who observed differences in how women and men were engaged in a mural design project. Men had different interests in the construction (e.g., how the tools were used) of the design of a distraction mural compared with women. Both articles suggest that more attention should be paid to gender and identity construction through the arts and activities in long-term care facilities, specifically among men living with dementia.

## Discussion and Implications

This review describes measures used to modulate the life-space mobility of people with dementia in residential care environments. Relying on such measures can be challenging and create moral issues where they are unreliable, dependent on resources, or not equitable or suited to the individual needs of all residents. In addition, using technology and outdoor spaces or opportunities to engage in activities beyond the care setting is often dependent on human resources. Therefore, residents of care homes with staff shortages, limited volunteer capacity, or no family support cannot experience measures that support their mobility and sense of freedom. Thus, long-term care providers need to balance the tensions between protecting physical safety, dignity and risk, paternalism, and respect for autonomy (Dreyfus et al., 2018).

To support the human rights of people with dementia, dementia care has to shift from a culture of restraints, control, and punitive safety measures to an inclusive, respectful, and innovative approach (Kontos et al., 2021). This new approach to dementia care needs to be based on connecting with people with dementia and understanding and responding to their needs (Huang et al., 2022). One step forward is to consider needs based on sex and gender differences. However, as demonstrated, research exploring the sex and gender differences of people with dementia continues to be neglected and ignored (Bartlett et al., 2018; Sandberg, 2018). Additionally, dementia care practices and built environments remain unchanged and continue to be gender-neutral or tailored to female residents’ needs. It could be argued that components of the residential care model are conventional and noncontemporary where they are not suited to women’s needs with changes in life-space levels, agency, and gender equality (i.e., more women working beyond the domestic environment; Bartlett et al., 2018). There is even less knowledge of the needs of diverse populations, including the LGBTQ+ community (Sandberg, 2018). This level of understanding would rejuvenate the current residential care system and contribute to new models of dementia care that reflect capacities based on individual interests and diverse backgrounds. This relates to the movement to focus on the capacity, individuality, and rights of persons with dementia, supporting a scenario of living well with dementia (Bellass et al., 2019; Vernooij-Dassen & Jeon, 2016).

One way to lead this shift is to question how measures used to limit mobility can be deceptive, patronizing, or override a resident’s capacity and dignity. As noted, residents can become frustrated when they know they are deceived (Mazzei et al., 2014; Øye & Jacobsen, 2020). Furthermore, care providers and others can recognize that a measure is fake while the resident may perceive it as real, contributing to paternalism and stigmatization (Huang et al., 2022; Lorey, 2019). This level of deception can be avoided by ensuring the measures are not too realistic and cause discomfort or boredom for residents (Lorey, 2019). In addition, environments should be designed to facilitate the mobility of residents with dementia (e.g., wayfinding) and stimulate a positive, socially inclusive environment that preserves the residents’ dignity (Tranvåg et al., 2013). Further studies exploring interactive, participatory design projects, such as the project described by Graham & Fabricius (2018), provide opportunities to design *with* people with dementia and will result in better residential care innovations and quality of life.

*Policy recommendations.—*Although increasing the mobility of people with dementia can increase the quality of life (Tufford et al., 2018), an open-door policy that balances the safety and autonomy of residents can be challenging. To do this effectively, residential care organizations need new regulations, resources, and training (Ciofi et al., 2022; Sandberg et al., 2022) according to different national contexts. Operating a caring environment can receive much public critique when the level of care is not optimal (Lowndes et al., 2021; Steele, Carr, et al., 2020; Steele, Swaffer, et al., 2020) due to inadequate staffing models, overworked and/or underpaid staff, and limited operational budgets (Heggestad et al., 2013; Øye et al., 2017). Such limitations can make it difficult to protect resident safety and promote quality of life without removing their rights to freedom (Steele, Swaffer, et al., 2020b). To help alleviate the level of risk and to reframe operational models that support the freedom of residents, policies, and regulations need to be updated, including an inspection process with a focus on less tangible measures, such as the resident’s daily experiences and quality of life, opposed to a focus on physical, measurable outcomes (Lowndes et al., 2021). To do so, more research is required to compare regulations and share best practices between care providers and countries.

Finally, it may be more difficult for some care facilities to open a door (when it used to be closed) than when the door is always open. One challenge could be the inaccessibility of the public spaces surrounding the care environment for older adults or environmental risks such as busy streets, roadways, and waterways (Aud, 2004; Evans et al., 2018; Øye and Jacobsen, 2020; Steele, Carr, et al., 2020). Such risks can result in staff determining that a closed-door environment is better for all residents. As described by Øye and Jacobsen (2020), busy roads resulted in staff hiding locks and closing doors and gates where they feared vulnerable residents were at risk if they were to leave the premises. As noted by Lowndes et al. (2021), an out-of-the-way location and physical space of a facility can lead to further isolation for those who do not receive visitors. Therefore, planners and policymakers should ensure that surrounding social and built environments are accessible for residents by identifying features in public spaces that can support or hinder the well-being of people with dementia (Sturge et al., 2021). For the built environment, planners should ensure well-maintained streets, modified traffic zones, identifiable landmarks, and clear signage. Additionally, the lack of community cohesion and support are barriers to realizing human rights (Steele, Swaffer, et al., 2020). Connecting care home residents to the community can be achieved through community development and education, for instance, Good Samaritan training for police and taxi drivers (Aud, 2004). A similar best practice is in place in Bruges, Belgium, where businesses with a logo of a red-knotted handkerchief are known as a place with trained staff who can respectfully respond and assist a person with dementia (Biggs & Carr, 2015). Other best practices that support mobility beyond the care environments include walking programs that allow people with dementia to gain familiarity with the neighborhood (MacAndrew et al., 2017) or a residents’ convoy (i.e., a network of family, staff, and volunteers) to promote accessibility of surrounding community resources and venues (Ciofi et al., 2022). Such community education and assistance programs should be adapted, implemented, and evaluated in other global community settings.

*Strengths and limitations.—*Although previous studies have looked at measures to limit the freedom of people with dementia, this is the first review focusing on measures used in the context of life space mobility in residential care facilities. Compared with earlier reviews by Neubauer et al. (2018) and Emrich-Mills et al. (2021), this scoping review has similar findings related to questioning the effectiveness and the ethical concerns of using measures to limit wandering behavior and mitigate the risk of exiting, eloping, or getting lost. Our study can also be compared with a recent systematic review of factors that influence freedom of movement on health among people with dementia living in nursing homes (van Liempd et al., 2022). The van Liempd et al. (2022) review identified 16 articles, seven of which have been identified in the present scoping review, referred to the positive health framework to deductively present the results and some of the findings are similar (e.g., the importance of gardens and access to meaningful activities) to our review. However, due to the combination of the scoping review framework, a double-blind screening tool to select articles, and an inductive approach to interpreting the results, our review has resulted in twice as many articles being included (16 vs 30 articles). Further research is needed to explore the factors influencing reviewers’ decisions and unintended bias.

A limitation of our review is that it is not representative of all people with dementia living in residential care facilities. We focused on life-space mobility outside the patient room and not on measures to limit patient mobility to a bedroom setting. Additionally, this review did not explore the risks of not using these measures, hence the need for more research. Although we used a systematic approach to the search, some relevant studies may have been missed. Moreover, there are a variety of operational models in dementia care that have not been explored in academic research. Furthermore, this review includes only residential care models in Western societies, and the findings cannot be generalized to other welfare states.

## Conclusion

Residential care environments use a range of measures to restrict and support the life-space mobility of residents with dementia. These measures were originally intended to secure the safety of people with dementia who are frequently disoriented and wander. However, relying on such measures does not always consider or acknowledge the human rights of people with dementia. As the human rights of persons with dementia become more realized, residential dementia care will need to be redesigned based on the individual needs, diversity, and capacities of residents with dementia. This culture shift will require new care structures with renewed policy directions to provide more dignified care within and beyond the boundaries of the care environment. New measures and strategies are needed to promote mobility instead of relying on strategies to restrain and modify the life spaces of people with dementia. Through person-centered, engaging, and innovative care solutions based on the needs of people with dementia, we can truly find the balance between the safety and freedom to support the quality of life of people with dementia.

## Supplementary Material

gnad071_suppl_Supplementary_Material

## Data Availability

This study was preregistered with Open Science Framework (https://osf.io/8cdq7). The systematic method, PRISMA statement, search terms, and search strategy facilitate the study replication (refer to Supplementary Materials). All data referred to in this review were secondary published data.
